# Supplementary material for: Modeling the influence of the hippocampal memory system on the oculomotor system
Source: Netw Neurosci. 2020 Mar 1;4(1):217–33. doi: 10.1162/netn_a_00120 (PMC7055646; doi:10.1162/netn_a_00120)
Supplement: Supplementary file 1 [file netn-04-217-s001.pdf]

Ryan, J. D., Shen, K., Kacollja, A., Tian, H, Griffiths, J., Bezgin, G., McIntosh, A. R. (2020). Supporting information for "Modelling the influence of the hippocampal memory system on the oculomotor system." *Network Neuroscience*, *4*(1), 217–233. https://doi.org/10.1162/netn_a_00120

Supplementary Materials

Modelling the influence of the hippocampal memory system on the oculomotor system

Jennifer D. Ryan*, Kelly Shen*, Arber Kacollja, Heather Tian, John Griffiths, Gleb Bezgin, Anthony R. McIntosh

** equal contribution*

Supplementary Table 1. Additional Generic 2D oscillator model parameters

Parameter	Value	Description
d	0.07674	Temporal scale factor
τ	1	Time-scale hierarchy parameter
f	1	Coefficient for fast variable cubic self-feedback term
e	0	Coefficient for fast variable quadratic self-feedback term
g	-0.1	Coefficient of fast variable linear self-feedback term
α	1	Coefficient for linear input term from slow to fast variable
γ	1	Additional scaling parameter for fast variable constant input *I* and long-range inputs
c	0	Coefficient for quadratic input term from fast variable to slow variable
b	-12.3038	Coefficient for linear input term from fast variable to slow variable
β	0	Coefficient for slow variable linear self-feedback term
a	0	Slow variable constant input term
I	0	Fast variable constant input term

Supplementary Table 2. Activation times (ms) following simulated stimulation of hippocampal subfields and medial temporal lobe regions for each node in the model. Observation nodes are presented in alphanumeric order. Observations nodes of interest noted in the main manuscript (HC/MTL regions, oculomotor nodes, and regions that are involved in the shortest paths between HC/MTL and oculomotor nodes) are bolded. S= subiculum, PrS = pre-subiculum, PaS=para-subiculum; ERC = entorhinal cortex; 35/36 = perirhinal cortex; TF/TH = parahippocampal cortex. *0* = stimulation onset; N/A = no response observed.

	**Stimulated Node**									
**Observation Node**	**CA3**	**CA1**	**S**	**PrS**	**PaS**	**ERC**	**35**	**36**	**TF**	**TH**
1	N/A	676	N/A	157	N/A	221	125	145	312	174
2	N/A	645	N/A	119	N/A	372	151	206	251	153
4	N/A	534	N/A	141	N/A	265	103	115	262	152
**5**	N/A	322	50	92	555	249	129	141	69	64
6	N/A	239	630	25	816	115	22	15	82	39
9	N/A	109	192	14	173	15	12	15	18	17
**10**	N/A	59	24	83	106	12	10	12	81	20
**11**	N/A	12	68	74	166	10	7	8	38	48
**12**	N/A	96	186	93	285	15	9	10	24	34
**13**	N/A	10	18	62	132	8	7	11	19	19
**14**	N/A	9	27	57	76	9	9	11	13	20
**23**	N/A	165	384	12	231	43	37	52	24	13
**24**	N/A	107	250	55	238	24	22	25	22	16
**25**	435	6	19	35	57	6	11	8	9	12
29	98	20	133	32	42	10	21	19	0	0
30	336	55	265	61	118	14	127	128	0	2
**32**	N/A	38	63	88	89	10	13	14	14	17
**35**	567	6	21	49	50	0	*0*	0	9	19
**36**	317	1	14	10	6	0	0	*0*	6	14
45	N/A	172	334	84	464	63	14	15	23	88
**46**	N/A	84	135	15	187	12	9	11	17	23
3a	N/A	631	N/A	143	N/A	409	89	124	292	190
3b	N/A	767	N/A	333	N/A	184	113	126	482	236
**7a**	N/A	381	202	336	795	256	53	52	103	59
7b	N/A	385	566	152	N/A	318	101	124	155	132
A1	N/A	N/A	N/A	N/A	N/A	48	18	24	N/A	64
AII	N/A	N/A	N/A	N/A	N/A	108	20	18	409	165
AITd	N/A	159	437	210	710	469	409	341	35	200
AITv	365	23	129	40	146	128	134	83	0	11
**CA1**	137	*0*	0	0	46	0	8	2	0	0
**CA3**	*0*	48	31	37	27	0	21	20	48	1
CITd	N/A	226	639	270	604	604	381	370	56	121
CITv	N/A	34	179	57	327	160	134	103	0	59
CM	N/A	N/A	N/A	N/A	N/A	136	40	40	805	83

	DP	N/A	362	N/A	355	678	368	172	173	95	105
	**ERC**	169	40	0	4	0	*0*	0	0	7	13
	**FEF**	N/A	217	452	68	500	79	19	15	34	71
	**FST**	186	47	147	52	79	6	1	3	0	6
	G	N/A	470	N/A	150	N/A	278	66	69	366	214
	Id	N/A	90	241	120	268	12	7	7	32	19
	**Ig**	N/A	11	63	75	189	11	4	6	21	13
	L	N/A	N/A	N/A	N/A	N/A	104	21	17	459	293
	**LIP**	N/A	535	N/A	445	N/A	561	233	230	176	144
	MDP	N/A	548	353	307	874	544	263	276	211	178
	**MIP**	N/A	237	523	215	296	405	113	126	70	48
	**MSTd**	N/A	286	664	287	494	33	23	23	94	53
	MSTl	N/A	145	329	151	238	19	11	12	49	29
	**MT**	N/A	260	857	272	483	189	107	23	36	83
	Pa	N/A	N/A	N/A	N/A	N/A	33	13	18	525	130
	PAC	60	43	41	42	37	0	0	0	49	4
	**PaS**	350	17	96	28	*0*	50	15	0	0	32
	PIP	N/A	250	743	228	315	454	134	147	76	52
	Pir	166	64	23	37	24	0	24	24	62	11
	PITd	N/A	217	637	226	360	460	153	171	52	64
	PITv	N/A	177	507	224	538	485	339	315	37	114
	**PO**	656	208	593	190	248	355	99	107	60	39
	**Proisocortex**	N/A	52	96	12	48	3	1	4	29	20
	Prostriata	N/A	N/A	N/A	N/A	N/A	N/A	N/A	N/A	N/A	N/A
	**PrS**	84	23	119	*0*	25	6	13	11	0	0
	Ri	N/A	534	N/A	702	N/A	208	68	82	351	249
	RL	N/A	267	N/A	N/A	N/A	38	9	8	457	34
	**S**	115	17	*0*	26	37	0	16	10	0	0
	SII	N/A	124	397	140	N/A	74	7	9	46	123
	SMA	N/A	377	641	153	957	174	139	118	172	98
	STPa	N/A	46	110	59	104	4	0	0	2	9
	STPp	N/A	252	N/A	369	N/A	89	10	8	75	74
	**TF**	137	0	20	0	44	11	12	6	*0*	0
	**TH**	7	0	29	0	0	20	29	18	0	*0*
	V1	N/A	217	670	197	261	399	117	125	64	40
	**V2**	215	56	274	48	66	139	16	17	6	0
	**V3**	N/A	235	733	224	313	389	126	137	60	52
	V3A	N/A	260	841	257	409	398	154	163	68	68
	**V4**	N/A	76	353	103	244	280	147	142	5	15
	V4t	N/A	273	936	280	495	369	175	149	66	88
	VIP	N/A	506	N/A	391	N/A	508	195	202	160	116
	VOT	N/A	231	665	245	401	489	177	194	57	75
	**VP**	753	55	266	86	259	223	98	101	2	41

Supplementary Table 3. Changes in activation times (ms) for nodes of interest following a lesion of CA1 and simulated stimulation of hippocampal subfields and medial temporal lobe regions. Values were determined by subtracting the intact stimulation times (Table 1) from lesioned activation times, such that slower responses are positive while faster responses are negative.

		Stimulated Node									
		**CA3**	**CA1**	**S**	**PrS**	**PaS**	**ERC**	**35**	**36**	**TF**	**TH**
**Observation Node**	**CA3**	*0*		-4	-2	0	0	0	0	-5	-1
	**CA1**										
	**S**	-10		*0*	-7	-4	0	-1	-1	0	0
	**PrS**	-4		-3	*0*	-1	-2	-1	-1	0	0
	**PaS**	-32		106	-7	*0*	-8	-1	0	0	-8
	**ERC**	-3		0	-1	0	*0*	0	0	-1	-1
	**35**	N/A		-1	0	0	0	*0*	0	-1	-3
	**36**	55		-2	-1	0	0	0	*0*	-1	-2
	**TF**	-19		214	0	-9	-3	-2	-1	*0*	0
	**TH**	0		327	0	0	-3	-4	-2	0	*0*
	**5**	N/A		1	0	2	0	0	-1	0	0
	**10**	N/A		-1	-6	1	0	0	0	-7	-1
	**11**	N/A		25	11	3	0	0	0	1	-3
	**12**	N/A		10	-6	3	0	0	1	-2	-2
	**13**	N/A		1	17	2	0	0	0	0	-1
	**14**	N/A		0	17	1	0	0	0	0	-1
	**23**	N/A		N/A	0	0	0	0	0	0	0
	**25**	63		-2	14	2	0	0	0	0	-1
	**32**	N/A		0	-4	1	0	0	0	0	0
	**Ig**	N/A		22	28	4	0	0	0	1	0
	**Pro**	N/A		3	0	1	1	0	0	0	0
	**7a**	N/A		-2	1	-1	-2	-1	-1	-1	0
	**FST**	-5		13	-3	-1	0	0	0	0	0
	**MIP**	N/A		N/A	-4	-1	4	-1	-1	-2	-1
	**PO**	-11		N/A	-6	-2	3	-2	-1	-2	-1
	**MSTd**	N/A		N/A	1	2	0	0	0	-1	0
	**MT**	N/A		N/A	0	-2	-1	-1	-1	-1	0
	**V4**	N/A		N/A	-4	-2	11	-2	-2	-1	-1
	**VP**	-19		N/A	-5	-3	8	-2	-2	0	-2
	**V3**	N/A		N/A	-5	-2	4	-1	-3	-2	0
	**V2**	-3		N/A	-4	-1	-1	0	0	-1	0
	**24**	N/A		33	-1	3	0	0	0	0	0
	**46**	N/A		1	0	2	0	0	0	-1	-1
	**FEF**	N/A		84	-3	5	0	0	0	-1	-1
	**LIP**	N/A		N/A	0	N/A	0	-2	-2	-3	-2

Supplementary Table 4. Changes in activation times (ms) for nodes of interest following a lesion of PrS and simulated stimulation of hippocampal subfields and medial temporal lobe regions. Conventions as in Supplementary Table 3.

	Stimulated Node									
Observation Node	CA3	CA1	S	PrS	PaS	ERC	35	36	TF	TH
CA3	*0*	-3	0		-1	0	0	0	-6	-1
CA1	-17	*0*	0		-9	0	-1	-2	0	0
S	-9	0	*0*		-3	0	-1	0	0	0
PrS										
PaS	-20	1	-3		*0*	-4	0	0	0	-8
ERC	38	-3	0		0	*0*	0	0	-1	-3
35	N/A	0	0		0	0	*0*	0	0	-3
36	38	0	0		0	0	0	*0*	-1	-2
TF	-14	0	1		-7	-2	-1	0	*0*	0
TH	-1	0	-4		0	-4	-5	-2	0	*0*
5	N/A	3	1		12	5	0	-1	0	0
10	N/A	-1	0		0	0	0	0	-5	-1
11	N/A	0	0		2	0	0	0	-2	-5
12	N/A	0	1		2	0	0	1	-1	-3
13	N/A	0	0		1	0	0	0	-1	-1
14	N/A	0	0		1	0	0	0	0	-1
23	N/A	4	1		5	0	-1	0	0	0
25	8	1	0		1	0	0	0	1	-1
32	N/A	0	0		0	0	0	0	0	0
Ig	N/A	0	1		2	0	0	0	1	0
Pro	N/A	0	1		1	1	0	0	0	0
7a	N/A	-2	0		1	-2	-1	-1	-1	-1
FST	-6	-1	-1		-1	0	0	0	0	0
MIP	N/A	-2	-5		-2	3	-2	-1	-1	2
PO	-13	-2	-6		-3	2	-2	-1	-1	-2
MSTd	N/A	0	4		2	0	0	0	0	-1
MT	N/A	-1	-9		-3	-1	-1	-1	0	-1
V4	N/A	-1	-3		-3	0	-2	-2	0	-1
VP	-16	-2	-3		-3	0	-2	-2	0	-3
V3	N/A	-2	-8		-3	0	-1	-3	-1	-1
V2	-4	-1	-4		-2	-1	0	-1	-1	0
24	N/A	1	3		3	1	0	0	0	-1
46	N/A	-1	1		5	0	0	0	-1	-1
FEF	N/A	0	4		18	0	0	0	0	-4
LIP	N/A	-4	N/A		N/A	-5	-2	-2	-3	-3

Supplementary Table 5. Changes in activation times (ms) for nodes of interest following a lesion of all hippocampal subfields and simulated stimulation of medial temporal lobe regions. Conventions as in Supplementary Table 3.

		Stimulated Node									
		**CA3**	**CA1**	**S**	**PrS**	**PaS**	**ERC**	**35**	**36**	**TF**	**TH**
**Observation Node**	**CA3**										
	**CA1**										
	**S**										
	**PrS**										
	**PaS**										
	**ERC**						*0*	0	0	-3	-4
	**35**						0	*0*	0	-1	-5
	**36**						0	0	*0*	-2	-3
	**TF**						-4	-3	-4	*0*	0
	**TH**						-6	-7	-4	0	*0*
	**5**						14	-1	-2	0	0
	**10**						0	0	0	-10	-2
	**11**						0	0	0	0	-7
	**12**						0	0	1	-3	-5
	**13**						0	0	0	-1	-2
	**14**						0	0	0	0	-2
	**23**						0	-1	0	0	-1
	**25**						0	0	0	0	-1
	**32**						0	0	0	-1	-1
	**Ig**						0	0	0	1	0
	**Pro**						1	0	0	0	-1
	**7a**						0	-1	-1	-2	-1
	**FST**						0	1	0	0	-1
	**MIP**						12	-3	-2	-2	-2
	**PO**						7	-3	-2	-3	-2
	**MSTd**						0	0	0	-2	-1
	**MT**						-2	-2	-1	-1	-1
	**V4**						14	-3	-3	-1	-1
	**VP**						8	-4	-3	-1	-3
	**V3**						7	-3	-5	-3	-2
	**V2**						-1	0	-1	-1	0
	**24**						1	0	0	0	-1
	**46**						0	0	0	-1	-2
	**FEF**						0	0	0	-2	-5
	**LIP**						1	-4	-4	-4	-4

Supplementary Table 6. Changes in activation times (ms) for nodes of interest following a lesion of the ERC and simulated stimulation of hippocampal subfields and medial temporal lobe regions. Conventions as in Supplementary Table 3.

		Stimulated Node									
		CA3	CA1	S	PrS	PaS	ERC	35	36	TF	TH
Observation Node	**CA3**	*0*	-8	127	-2	6		54	51	-6	-1
	**CA1**	-1	*0*	0	0	0		-1	-2	0	0
	**S**	-3	0	*0*	-2	2		-2	-1	0	0
	**PrS**	0	0	14	*0*	-1		-1	-1	0	0
	**PaS**	-3	0	0	0	*0*		-1	0	0	0
	**ERC**										
	**35**	-22	0	-1	4	34		*0*	0	-1	-3
	**36**	-17	-1	-2	-1	0		0	*0*	-1	-1
	**TF**	-1	0	1	0	-1		-1	0	*0*	0
	**TH**	0	0	0	0	0		-2	-1	0	*0*
	**5**	N/A	0	0	0	1		-2	-4	0	0
	**10**	N/A	-1	-1	4	75		-1	-1	-2	0
	**11**	N/A	0	15	2	139		-1	-1	-1	-2
	**12**	N/A	-1	107	-2	267		0	0	-1	-1
	**13**	N/A	0	1	3	108		0	-1	0	-1
	**14**	N/A	0	1	3	104		-1	-1	0	-1
	**23**	N/A	0	-5	0	1		-1	-1	0	0
	**25**	26	0	-3	0	45		-1	-1	0	-1
	**32**	N/A	0	70	20	86		-1	-1	0	0
	**Ig**	N/A	0	2	2	28		0	-1	0	0
	**Pro**	N/A	0	98	0	1		-1	-1	0	0
	**7a**	N/A	-1	-2	0	22		-1	0	0	0
	**FST**	-1	-1	78	-1	3		-1	-1	0	0
	**MIP**	N/A	0	-6	0	0		-1	0	0	0
	**PO**	0	0	-10	0	0		-1	0	0	0
	**MSTd**	N/A	0	N/A	8	46		0	0	0	0
	**MT**	N/A	0	72	-1	7		0	0	0	0
	**V4**	N/A	0	-3	0	0		0	0	0	0
	**VP**	-1	0	-2	0	0		-1	-1	0	0
	**V3**	N/A	0	-17	0	0		0	-1	0	0
	**V2**	0	0	-4	0	0		0	0	0	0
	**24**	N/A	-1	96	-1	29		0	-1	0	-1
	**46**	N/A	-2	101	0	64		0	0	0	0
	**FEF**	N/A	-3	342	-2	106		-1	0	0	-1
	**LIP**	N/A	-1	N/A	-1	N/A		-1	-1	-1	0

Supplementary Table 7. Changes in activation times (ms) for nodes of interest following a combined lesion of areas TH and TF and simulated stimulation of hippocampal subfields and medial temporal lobe regions. Conventions as in Supplementary Table 3.

		Stimulated Node									
		CA3	CA1	S	PrS	PaS	ERC	35	36	TF	TH
Observation Node	**CA3**	*0*	115	-3	-1	-4	0	0	1		
	**CA1**	N/A	*0*	0	0	45	0	-1	-2		
	**S**	N/A	120	*0*	17	-5	0	-4	-2		
	**PrS**	N/A	185	-29	*0*	101	-6	-3	-2		
	**PaS**	N/A	23	16	33	*0*	-16	-4	0		
	**ERC**	N/A	-7	0	-3	0	*0*	0	0		
	**35**	N/A	0	0	-5	1	0	*0*	0		
	**36**	N/A	-1	-1	-1	0	0	0	*0*		
	**TF**										
	**TH**										
	**5**	N/A	N/A	2	1	N/A	3	-1	-3		
	**10**	N/A	-4	-1	-4	10	0	0	0		
	**11**	N/A	0	0	-7	4	0	0	0		
	**12**	N/A	1	1	-5	35	0	0	1		
	**13**	N/A	0	1	-3	11	0	0	0		
	**14**	N/A	0	1	-5	3	0	0	0		
	**23**	N/A	N/A	N/A	0	N/A	-1	-1	0		
	**25**	N/A	0	0	-1	6	0	0	0		
	**32**	N/A	-1	0	11	6	0	0	0		
	**Ig**	N/A	1	3	31	142	0	0	0		
	**Pro**	N/A	0	1	0	2	1	0	0		
	**7a**	N/A	N/A	5	N/A	N/A	-4	-2	-2		
	**FST**	N/A	68	12	96	130	0	2	1		
	**MIP**	N/A	N/A	N/A	349	N/A	27	-8	-8		
	**PO**	N/A	N/A	N/A	N/A	N/A	19	-9	-7		
	**MSTd**	N/A	N/A	N/A	N/A	N/A	1	0	0		
	**MT**	N/A	N/A	N/A	N/A	N/A	-4	-4	-1		
	**V4**	N/A	N/A	N/A	N/A	N/A	67	-7	0		
	**VP**	N/A	N/A	N/A	N/A	N/A	-4	-4	-1		
	**V3**	N/A	N/A	N/A	N/A	N/A	17	-10	-11		
	**V2**	N/A	N/A	N/A	N/A	N/A	3	-1	-1		
	**24**	N/A	46	21	3	412	1	1	0		
	**46**	N/A	7	1	-1	53	0	0	0		
	**FEF**	N/A	69	35	-5	N/A	1	0	0		
	**LIP**	N/A	N/A	N/A	N/A	N/A	1	-9	-8		

Supplementary Table 8. Changes in activation times (ms) for nodes of interest following a combined lesion of areas 35 and 36 and simulated stimulation of hippocampal subfields and medial temporal lobe regions. Conventions as in Supplementary Table 3.

		Stimulated Node									
		CA3	CA1	S	PrS	PaS	ERC	35	36	TF	TH
Observation Node	**CA3**	*0*	-5	-3	-1	-2	0			-3	0
	**CA1**	-2	*0*	0	0	-1	0			0	0
	**S**	-3	0	*0*	-1	-1	0			0	0
	**PrS**	0	0	-1	*0*	0	-2			0	0
	**PaS**	-22	1	-7	0	*0*	34			0	0
	**ERC**	-19	19	0	-3	0	*0*			-2	-2
	**35**										
	**36**										
	**TF**	-1	0	0	0	-1	-1			*0*	0
	**TH**	0	0	0	0	0	-2			0	*0*
	**5**	N/A	-1	0	-1	-5	18			0	0
	**10**	N/A	5	0	-3	22	-2			5	-1
	**11**	N/A	0	5	1	87	-2			1	-3
	**12**	N/A	17	26	-1	158	-3			-2	-2
	**13**	N/A	0	1	-1	23	-1			0	-1
	**14**	N/A	0	1	-3	13	-1			0	-1
	**23**	N/A	0	-3	0	-1	-2			0	0
	**25**	125	0	-1	-1	7	-1			1	-1
	**32**	N/A	1	3	-4	14	-1			0	0
	**Ig**	N/A	0	-4	1	45	-1			0	-1
	**Pro**	N/A	7	8	0	2	0			1	0
	**7a**	N/A	15	-2	-1	17	93			0	0
	**FST**	-9	-2	2	-2	-1	-1			0	0
	**MIP**	N/A	0	-5	0	1	173			0	0
	**PO**	5	0	-10	1	1	158			0	0
	**MSTd**	N/A	43	285	2	32	-1			-1	-1
	**MT**	N/A	3	48	-1	10	42			0	0
	**V4**	N/A	1	2	1	2	19			0	0
	**VP**	9	0	1	1	1	16			0	0
	**V3**	N/A	1	-15	1	1	142			0	0
	**V2**	1	0	-5	0	0	81			0	0
	**24**	N/A	1	6	-1	6	-2			0	-1
	**46**	N/A	14	11	0	52	-1			-1	-1
	**FEF**	N/A	53	90	-2	83	-3			-2	0
	**LIP**	N/A	4	N/A	-3	N/A	N/A			-1	-1

Supplementary Table 9. Changes in activation times (ms) for nodes of interest following a combined lesion of areas V4, 7a, 5 and 23 and **simulated** **stimulation of hippocampal subfields and medial temporal lobe regions.** Conventions as in Supplementary Table 3.

	Stimulated Node									
Observation Node	CA3	CA1	S	PrS	PaS	ERC	35	36	TF	TH
CA3	*0*	0	0	0	1	0	0	1	1	0
CA1	-57	*0*	0	0	-6	1	-1	1	0	0
S	-43	0	*0*	0	-2	0	-1	0	0	0
PrS	-31	-1	-35	*0*	-2	-1	-3	-2	0	0
PaS	-94	0	-4	0	*0*	-3	-1	0	0	0
ERC	43	2	0	0	0	*0*	0	0	0	0
35	N/A	1	2	2	8	0	*0*	0	1	0
36	118	2	1	0	1	0	0	*0*	0	1
TF	-54	0	1	0	-4	-1	-2	0	*0*	0
TH	-2	0	-3	0	0	-4	-10	-4	0	*0*
5										
10	N/A	0	0	-2	1	0	0	0	0	0
11	N/A	0	1	0	10	0	0	0	3	-1
12	N/A	5	12	-1	37	1	0	1	2	1
13	N/A	1	1	-1	2	0	0	0	0	0
14	N/A	1	0	-2	-2	0	0	0	0	0
23										
25	-57	1	1	0	3	0	0	0	1	0
32	N/A	0	-2	-5	-4	0	0	0	0	0
Ig	N/A	0	-4	-2	11	1	1	0	0	-1
Pro	N/A	2	8	1	8	1	-1	0	4	1
7a										
FST	-12	7	7	1	1	1	4	2	3	1
MIP	N/A	-57	-69	-39	-94	-191	-55	-65	-11	-4
PO	-320	-36	-142	-25	-54	-165	-47	-51	-7	-1
MSTd	N/A	-58	169	-58	-47	-12	-6	-5	-31	-19
MT	N/A	-39	-118	-53	-102	-86	-48	-9	-11	-18
V4										
VP	-432	-16	-75	-22	-77	-113	-53	-51	-2	-8
V3	N/A	-49	-219	-38	-81	-200	-65	-72	-12	-5
V2	-101	-11	-59	-7	-14	-68	-5	-6	-1	0
24	N/A	-12	-5	11	-24	-2	-1	-2	-2	-2
46	N/A	8	24	1	48	1	1	1	2	1
FEF	N/A	48	124	7	187	9	2	3	10	6
LIP	N/A	-273	N/A	-197	N/A	-297	-109	-104	-120	-79
